# Association between *hTERT* polymorphisms and the risk of breast cancer in a sample of Southeast Iranian population

**DOI:** 10.1186/1756-0500-7-895

**Published:** 2014-12-10

**Authors:** Mohammad Hashemi, Shadi Amininia, Mahboubeh Ebrahimi, Seyed Mehdi Hashemi, Mohsen Taheri, Saeid Ghavami

**Affiliations:** Cellular and Molecular Research Center, Zahedan University of Medical Sciences, Zahedan, Iran; Department of Clinical Biochemistry, School of Medicine, Zahedan University of Medical Sciences, Zahedan, Iran; Department of Internal Medicine, School of Medicine, Zahedan University of Medical Sciences, Zahedan, Iran; Genetics of Non-communicable Disease Research Center, Zahedan University of Medical Sciences, Zahedan, Iran; Department of Human Anatomy and Cell Science, College of Medicine, Faculty of Health Science, Manitoba Institute of Child Health, University of Manitoba, Winnipeg, Manitoba Canada; Health Policy Research Centre, Shiraz Medical University, Shiraz, Iran

**Keywords:** *hTERT*, Breast cancer, Polymorphism, Genotyping, MNS16A

## Abstract

**Background:**

Breast cancer (BC) is considered to be one of the most important causes of death worldwide, and it affects the Iranian female population a decade earlier than female in other parts of the world. Human telomerase reverse transcriptase (*hTERT*) is a main subunit of the telomerase complex. MNS16A is located downstream of the *hTERT* gene and is recognized as the regulator of *hTERT* promoter activity. The aim of the present study was to access the possible impact of *hTERT* variants on BC risk in an Iranian population in southeast Iran.

**Methods:**

A total of 491 subjects including 266 BC patients and 225 healthy women participated in the study. Polymerase chain reaction (PCR) was used to genotype the MNS16A variable number of tandem repeats and 177 bp ins/del polymorphisms in the *hTERT* gene. PCR-RFLP and ARMS-PCR were used to genotype *hTERT* rs2736098 and 2735940, respectively. The association between genotypes and BC was assessed by computing the odds ratio (OR) and 95% confidence intervals (95% CI) from logistic regression analyses. A p-value of <0.05 was considered statistically significant.

**Results:**

The MNS16A genotype frequency distribution in BC patients was: LL, 43.2%; LS, 51.1%; and SS, 5.7%, and in controls: LL, 29.5%; LS, 68.3%; and SS, 2.2%. The LS genotype decreased the risk of BC compared with LL (OR = 0.51, 95% CI = 0.35-0.75, p < 0.001). The *hTERT* 177 bp ins/del polymorphism was not polymorphic in our population. All subjects had the ins/ins genotype. Our findings indicate that the MNS16A genotype and *hTERT* rs2736098 variant were associated with BC risk in the study. We also showed that the rs2736098 A/G polymorphism increased the risk of BC (OR = 1.80, 95% CI = 1.12-2.88, p = 0.017, AG vs AA; OR = 1.80, 95% CI = 1.06-3.06, p = 0.033, GG vs AA; OR = 1.87, 95% CI = 1.19-2.94, p = 0.006, AG + GG vs AA). No significant association was found between the rs2735940 C/T variant and BC.

**Conclusion:**

Our findings indicate that the MNS16A genotype and the *hTERT* rs2736098 variant influence the risk of BC in an Iranian population in southeast Iran.

## Background

Many women are diagnosed with breast cancer (BC) each day worldwide. Globally, BC may be considered the most common cancer among females and it is also the leading cause of cancer-related deaths in many countries [1]. BC is recognized as an important health care problem worldwide, affecting approximately 1 million women annually [1–3]. BC is also reported to be one of the most frequent malignancies among Iranian women, and it comprises 21.4% of female cancers in this population [4]. Interestingly, it has been reported that BC affects Iranian women about a decade earlier than women in Western countries [5], which highlights the importance of research on BC in the Iranian population. Several different factors are involved in BC pathogenesis, but its exact etiology is complicated and is not clearly identified. Our previous investigations provided solid evidence that genetic factors play important roles in the pathogenesis and progression of this malignancy in the population in southeast Iran [6–11].

Telomeres are repeat sequences of TTAGGG at the end of linear chromosomes and are responsible for protecting against loss of genetic information during the process of cellular division [12, 13]. Repeated cell cycles cause telomere shortening, drive the cells into the senescence condition, and finally trigger programmed cell death I, which potentially protects the cells against genomic instability and carcinogenesis [14]. Therefore, telomeres could be considered to be a key factors in cellular genomic maintenance and a potential candidate for carcinogenesis [12]. Human telomerase reverse transcriptase (hTERT) is a catalytic subunit of the telomerase [15], which together with the telomerase RNA component (TERC), is the main subunit of the telomerase complex [16]. Human TERT (hTERT) is located in 5p15.33 [17] and consists of 16 exons. Telomerase is a ribonucleoprotein that maintains integrity in the telomere regions, which subsequently shorten each replication cycle [18, 19]. Without telomeres, genomes would increasingly lose their information and would be truncated after cell division because enzymes that replicate DNA cannot continue duplication all the way to the chromosome ends [20]. It has been proposed that genetic instability is a driving force for transformation of normal cells into malignant cells during carcinogenesis [21].

MNS16A is known as a polymorphic tandem repeat minisatellite that is located downstream of the *hTERT* gene and it was first shown to be involved in promoter activity in lung cancer cell lines [22]. The variants that contain short tandem repeats have more effective promoter activity than those with long repeats, highlighting the importance of the number of tandem repeats in the risk of lung cancer. Many other groups have investigated the role of MNS16A in the etiology of different malignancies including cerebral [23], lung [24], breast [25], and colorectal cancer [26], but their results were inconsistent.

Because *hTERT* is the key molecular complex that maintains telomere stability, genetic variants in *hTERT* might impact on the risk of BC. However, considering the important role of MNS16A in *hTERT* gene promoter activity, we evaluated the MNS16A genotype and the impact of *hTERT* polymorphisms on BC susceptibility in a sample of the Iranian population.

## Methods

### Patients

This case-control study enrolled 266 pathologically confirmed BC patients who were referred to the Ali Ebneh Abitaleb hospital (Iran) and 225 age- and population-matched healthy women who participated in a screening project for metabolic syndrome; they were unrelated to the patients and had no history of any type of cancer. The clinicopathologic characteristics of the patients are summarized in Table 1. Ethical approvals for recruitment were obtained from the local Ethics Committee of Zahedan University of Medical Sciences, and informed consent was obtained from all patients and healthy individuals. Blood samples from patients and healthy controls were collected in EDTA-containing tubes and DNA was extracted using the salting out method, as described previously [27]. The quality of the isolated DNA was verified using electrophoresis on 1% agarose gel, quantitated spectrophotometrically and stored at -20°C until further use.

**Table 1:** **Clinical and pathological characteristics of breast cancer patients**

Characteristics	Patients n (%)
Age (years)	
≤50	148 (55.6)
>50	111 (41.7)
Unknown	7 (2.6)
Pathological type	
Ductal	175 (65.8)
Others	91 (34.2)
Tumor Size (cm)	
≤ 2	88 (33.1)
>2	163 (61.3)
Unknown	15 (5.6)
Histological grade	
I	46 (17.3)
II	138 (51.9)
III	45 (16.9)
IV	1 (0.4)
Unknown	36 (13.5)
Stage	
I	44 (16.5)
II	99 (37.2)
III	70 (26.3)
IV	39 (14.7)
Unknown	14 (5.3)
Estrogen receptor	
Positive	154 (57.9)
Negative	83 (31.2)
Unknown	29 (10.9)
Progesterone Receptor	
Positive	147 (55.3)
Negative	88 (33.1)
Unknown	31 (11.6)
HER2 status	
Positive	128 (48.1)
Negative	124 (46.6)
Unknown	14 (5.3)

### Polymerase chain reaction

**Figure 1:**
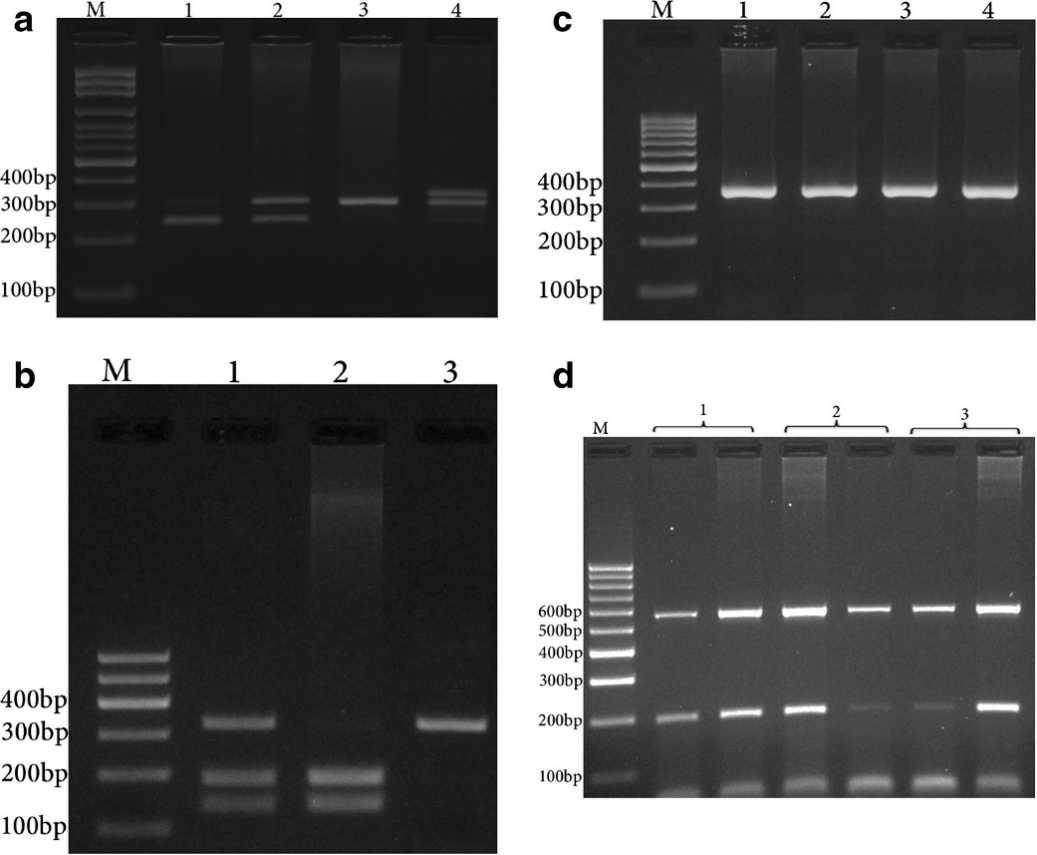
**Photograph of DNA electophoresis for detection of** ***hTERT*** **polymorphisms. a**: MNS16A. Lane 1, SS; lane 2, L/S; lanes 3 and 4, LL. **b**: rs1718119. Lane 1, GA; Lane 2, GG; lane 3, AA. **c**: *hTERT* 177 bp ins/del polymorphism. Lanes 1, 2, 3 and 4, ins/ins. **d**: rs2735940. Lane 1, TC; lane 2 TT; lane 3, CC. M = DNA marker.

Polymerase chain reaction (PCR) was used to genotype the MNS16A variable number of tandem repeat polymorphisms with the primer set, as previously reported [22]. The forward and reverse primer sequences were 5′-AGGATTCTGATCTCTGAAGGGTG-3′ and 5′-TCTGCCTGAGGAAGGACGTATG-3′, respectively. PCR was performed using 2X Prime Taq Premix (Genet Bio, Korea). The amplification procedure consisted of an initial denaturing step for 5 min at 95°C followed by 30 cycles for 30 s at 95°C, 20 s at 67.5°C, and 17 s at 72°C, as well as a final extension step for 10 min at 72°C. The PCR products were visualized on 3% agarose gel containing 0.5 μg/ml of ethidium bromide (Figure 1a) and genotypes were assigned as previously reported [22]: the 243 bp and 272 bp bands were classified as the short (S*) allele, and the 333 bp and 302 bp bands were classified as the long (L*) allele, thus defining the MNS16A genotypes as L*/L*, L*/S* and S*/S*.

hTERT 2736098 genotyping was accomplished using RFLP. The forward and reverse primers were 5′AGGACGCGTGGACCGAGTGA-3′ and 5′- GGAACCCAGAAAGATGGTCTC-3′, respectively. In each 0.20 ml reaction, 1 μl of genomic DNA (~100 ng/ml), 1 μl of each primer and 10 μl of 2X Prime Taq Premix (Genet Bio, Korea) and 7 μl ddH_2_O were added. The PCR conditions were set as follows: 95°C for 5 min, 30 cycles of 95°C for 30 s, 67°C for 30 s, and 72°C for 28 s and a final extension step of 72°C for 10 min. The PCR product (10 μl) was digested using Bsp120I restriction enzyme. The G allele was digested and produced 137 bp and 187 bp fragments while the A allele was undigested and produced a 324 bp fragment (Figure 1b).

*hTERT* 177 bp insertion/deletion genotyping was performed using PCR with forward (5′-GACCATCCTGGACTGATGGC-3′) and reverse (5′-AGGGGTGAACAATGGCGAAT-3′) primers, which can produce 366 bp and 189 bp insertion and deletion alleles, respectively. The PCR cycling conditions were 95°C for 5 min, 30 cycles of 95°C for 30 s, 66°C for 30 s, and 72°C for 26 s and a final extension step of 72°C for 10 min. The PCR products were verified on 2% agarose gels containing 0.5 μg/ml ethidium bromide, and observed under UV light (Figure 1c).

The *hTERT* rs2735940 polymorphism was genotyped using the amplification refractory mutation system polymerase chain reaction (ARMS-PCR) method. The primer sequences were as follows: generic primer, 5′-CGGCAAACACTGAAATGCTA-3′; T allele, 5′-GGGATTTCTAGAAGAGCGACAT-3′; and C allele, 5′-GGGATTTCTAGAAGAGCGACAC-3′. The product size for the allele was 197 bp. Beta-2 microglobulin (B2MF: 5′-TGTAAACACTTGGTGCCTGATATAGCTTGA-3′, B2MR: 5′-CATCAGTATCTCAGCAGGTGCCACTAATCT-3′), which produces 574 bp fragments, was used as an internal control.

In each 0.20 ml reaction solution, 1.3 μl of genomic DNA (~100 ng/ml), 0.5 μl of each primer and 10 μl of 2X Prime Taq Premix (Genet Bio, Korea) and 6.7 μl ddH_2_O were added. The PCR cycling condition was an initial denaturation at 95°C for 5 min followed by 30 cycles of 30 s at 95°C, annealing temperature for 23 s at 60°C, and 30 s at 72°C, with a final extension of 72°C for 10 min. The PCR products were verified on 2% agarose gels containing 0.5 μg/ml ethidium bromide, and observed under UV light (Figure 1d). Product sizes were 252 bp for either of the Ins or Del alleles, and 574 bp for the internal control. The presence of a 252 bp allele-specific band, in conjunction with a 574 bp control band, was considered to be positive evidence for each particular allele. The absence of an allele-specific band and the presence of a control band were considered to be evidence for the absence of an allele (Figure 1d).

### Statistical analysis

Statistical analysis was performed using the statistical software package SPSS 18. The association between genotypes and BC were assessed by computing the odds ratio (OR) and 95% confidence intervals (95% CI) from logistic regression analyses. Haplotype analysis was performed using SNPStats software [28, 29]. The Hardy–Weinberg equilibrium was tested for polymorphisms. A p-value of <0.05 was considered statistically significant.

## Results and discussion

### Results

The study groups included 266 BC patients with a mean age of 48.9 ± 11.1 years and 225 healthy women with a mean age of 50.0 ± 12.9 years. The patient group demographic information is summarized in Table 1. No significant difference in age was found between the groups (p = 0.306). The frequency distribution of the MNS16A genotypes in BC patients was: LL, 43.2%; LS, 51.1%; and SS, 5.7%, and the distribution in controls was: LL, 29.5%; LS, 68.3%; and SS, 2.2% (Table 2). Our finding showed that the L/S and L/S + S/S decreased the risk of BC (OR = 0.51, 95% CI = 0.35-0.75, p < 0.001 and OR = 0.55, 95% CI = 0.38-0.81, p = 0.002, respectively) compared with the L/L genotype.

**Table 2:** **The genotypes and allele distribution of** ***hTERT*** **variants in breast cancer patients and the control group**

Variants	Patients n (%)	Controls n (%)	OR (95% CI)	p
MNS16A Genotype				
L/L	115 (43.2)	66 (29.5)	1.00	-
L/S	136(51.1)	153(68.3)	0.51 (0.35-0.75)	<0.001
S/S	15 (5.7)	5 (2.2)	1.72 (0.61-4.95)	0.460
L/S + S/S	151 (56.8)	158 (65.5)	0.55 (0.38-0.81)	0.002
Allele				
L	366 (68.8)	285 (63.6)	1.00	-
S	166 (31.2)	163 (36.7)	0.79 (0.61-1.03)	0.090
rs2736098				
AA	40 (15.8)	58 (26.1)	1.00	-
AG	140 (55.3)	113 (50.9)	1.80 (1.12-2.88)	0.017
GG	72 (28.5)	51 (23.0)	1.80 (1.06-3.06)	0.033
AG + GG	212 (83.8)	164 (73.9)	1.87 (1.19-2.94)	0.006
Allele				
A	220 (43.7)	229 (51.6)	1.00	-
G	284 (56.3)	215 (48.4)	1.38 (1.06-1.78)	0.016
rs2735940				
CC	45 (17.0)	39 (17.3)	1.00	-
CT	124 (47.0)	138 (61.3)	0.78 (0.48-1.28)	0.380
TT	95 (36.0)	48 (21.3)	1.72 (0.99-2.98	0.066
CT + TT	219 (83.0)	186 (82.6)	1.02 (0.64-1.64)	0.991
Allele				
C	214 (40.5)	216 (48.0)	1.00	-
T	314 (59.5)	234 (52.0)	1.35 (1.05-1.75)	0.020

The *hTERT* rs2736098 A/G variant was associated with BC risk (Table 2). Our results indicated that AG as well as GG and AG + GG increased the risk of BC (OR = 1.80, 95% CI = 1.12-2.88, p = 0.017; OR = 1.80, 95%CI = 1.06-3.06, p = 0.033 and OR = 1.87, 95% CI = 1.19-2.94, p = 0.006, respectively) compared with the AA genotype. The rs2736098 G allele increased the risk of BC compared with the A allele (OR = 1.38, 95% CI = 1.06-1.78, p = 0.016). Our results also demonstrated that the *hTERT* rs2735940 polymorphism was not associated with BC risk/protection, while the rs2735940 T allele increased the risk of BC compared with the C allele (OR = 1.35, 95% CI = 1.05-1.75, p = 0.020). The 177 bp ins/del polymorphism was not polymorphic in our population so that all patients and controls had the insertion allele for the *hTERT* 177 bp ins/del polymorphism.

Haplotype analysis is shown in Table 3. Haplotypes LCA and STA decreased the risk of BC compared with LTG (MNS16A T/rs2735940 T/rs2736098 G). No significant association was observed among the *hTERT* polymorphisms and clinicopathologic parameters, including tumor stage, tumor grade, estrogen and progesterone receptors (ER, PgR), tumor size, and human growth factor receptor 2 (HER2) (Table 4).

**Table 3:** **MNS16A, rs2735940 and rs2736098 haplotype frequencies of** ***hTERT*** **polymorphisms in breast cancer patients and the control group**

MNS16A	rs2735940	rs2736098	Patient	Control	OR (95% CI)	p
L	T	G	0.2316	0.1423	1.00	-
L	C	G	0.1638	0.2163	0.56 (0.22-1.42	0.220
L	T	A	0.1862	0.1259	0.92 (0.46-1.86)	0.810
L	C	A	0.1064	0.1514	0.50 (0.25-0.99)	0.048
S	T	A	0.1644	0.1047	0.35 (0.16-0.72)	0.005
S	T	G	0.0975	0.0875	0.78 (0.23-2.61)	0.680
S	C	A	0.0630	0.0739	0.41 (0.13-1.26)	0.120
S	C	G	0.0720	0.0385	1.00 (0.18-5.68)	0.970

**Table 4:** **Association between** ***hTERT*** **polymorphisms and clinicopathological characteristics**

Variables	MNS16A			p	rs2735940			p	rs2736098			p
	LL	LS	SS		CC	CT	TT		AA	AG	GG	
Age (years)				0.123				0.181				0.893
≤50	58	83	7		21	70	57		21	82	39	
>50	56	48	7		24	52	33		17	57	30	
Pathological type				0.812				0.704				
Ductal	78	87	10		30	85	60		30	91	45	0.351
Others	37	49	5		15	39	35		10	49	28	
Tumor size (cm)				0.429				0.427				0.575
≤2	35	47	6		12	44	32		14	48	19	
>2	77	79	7		33	76	54		22	89	46	
TNM Stage				0.850				0.237				0.595
I	19	22	3		7	25	12		8	27	8	
II	47	48	5		19	41	40		12	51	32	
III	32	35	3		14	38	17		12	37	17	
IV	13	24	2		5	17	17		4	21	11	
Grade				0.437				0.072				0.286
I	23	21	2		10	19	17		10	20	14	
II	64	67	7		18	62	58		17	81	34	
III	14	30	1		11	26	8		6	22	15	
IV	0	0	0		0	1	0		0	0	0	
ER status				0.858				0.090				0.260
Positive	68	78	8		26	81	47		23	79	46	
Negative	37	43	3		15	32	36		14	47	16	
PgR status				0.627				0.620				0.137
Positive	62	79	6		28	71	48		18	80	42	
Negative	41	42	5		13	42	33		19	44	20	
HER2 status				0.155				0.404				0.186
Positive	50	69	9		27	59	42		15	75	32	
Negative	61	59	4		18	60	45		22	59	36	

### Discussion

Telomeres are involved in maintaining genomic stability [30]. In the current study, we investigated the impact of *hTERT* variants on BC risk in a sample of the Iranian population in southeast Iran. Our data demonstrated that MNS16A LS and L/S + S/S decreased the risk of BC. AG as well as GG and AG + GG increased the risk of BC for the rs2736098 A/G polymorphism. The rs2736098 G allele was associated with an increased risk of BC. Although the *hTERT* rs2735940 C/T polymorphism was not associated with BC risk/protection, the rs2735940 T allele was significantly associated with BC risk. The 177 bp ins/del polymorphism was not polymorphic in our population (all individuals were the ins/ins genotype).

Earlier studies showed that *hTERT* mRNA expression is regulated by MNS16A in lung cancer [31], while studies in BC patients showed that MNS16A and BC risk association are strongly related to the geographic area of the study and the selection of the patient population [25, 32]. Glioblastoma multiforme studies have also confirmed that the MNS16A association with the risk of cancer incidence is highly dependent on the population’s ethnicity [23, 33, 34]. Overall, it can be concluded that there is much controversy regarding the association of MNS16A with different cancers, which highlights the importance of cancer origin and ethnicity in the results.

Studies on the *hTERT* rs2736098 variant also showed a significant controversy in association of the variant and BC risk [35–37]. Haiman et al. [37] observed a positive association between the 5p15 locus and the increased risk of BC while Savage et al. [35] suggested a protective effect of three correlated SNPs in this region, including rs2736098, among Polish women with a positive family history. For familial cancers, association was also observed, although not statistically significant, after Bonferroni adjustment. In the present study, we found that the rs2736098 polymorphism increased the risk of BC in our population, and it can be concluded that an association between BC risk and *hTERT* rs2736098 variant is generally related to ethnicity of the study population and the geographical location of the sample.

A functional variant located in the promoter of the *hTERT* gene, -1327C > T (rs2735940), is associated with telomere length [38]. There are few reports about the correlation between the *hTERT* rs2735940 variant and BC. Recently, Pellatt et al. found no association between the *hTERT* rs2735940 polymorphism and BC risk. They found that this variant was associated with estrogen receptor negative/progesterone receptor positive (ER-/PR-) tumors (OR = 0.73, 95% CI = 0.59-0.91) [39].

## Conclusion

Our findings indicate that the MNS16A genotype and the *hTERT* rs2736098 variant influence the risk of BC in an Iranian population in southeast Iran. A limitation of this study is the relatively small sample size. Further research on *hTERT* polymorphisms is required to validate our findings in other ethnic groups in the Iranian population and in Middle Eastern countries. Because BC is a prevalent disease among the female population worldwide, identifying potential markers that can identify the possibility of this cancer is of significant importance in identifying BC in these patients.

## Abbreviations

BC: Breast cancer
PCR: Polymerase chain reaction
hTERT: Human telomerase reverse transcriptase
ER: Estrogen receptor
PgR: Progesterone receptor
HER2: Human epidermal growth factor 2 receptor.
